# Risk of Diabetes Mellitus on Incidence of Out-of-Hospital Cardiac Arrests: A Case-Control Study

**DOI:** 10.1371/journal.pone.0154245

**Published:** 2016-04-22

**Authors:** Young Sun Ro, Sang Do Shin, Kyoung Jun Song, Joo Yeong Kim, Eui Jung Lee, Yu Jin Lee, Ki Ok Ahn, Ki Jeong Hong, Cardiac Arrest Pursuit Trial with Unique Registry and Epidemiologic Surveillance (CAPTURES) investigators

**Affiliations:** 1 Laboratory of Emergency Medical Services, Seoul National University Hospital Biomedical Research Institute, Seoul, Republic of Korea; 2 Department of Emergency Medicine, Seoul National University College of Medicine, Seoul, Republic of Korea; 3 Department of Emergency Medicine, Korea University Ansan Hospital, Ansan, Republic of Korea; 4 Department of Emergency Medicine, Seoul National University Boramae Medical Center, Seoul, Republic of Korea; Kaohsiung Chang Gung Memorial Hospital, TAIWAN

## Abstract

**Background:**

This study aimed to determine the risk of diabetes mellitus (DM) on incidence of out-of-hospital cardiac arrest (OHCA) and to investigate whether difference in effects of DM between therapeutic methods was observed.

**Methods:**

This study was a case-control study using the Cardiac Arrest Pursuit Trial with Unique Registration and Epidemiologic Surveillance (CAPTURES) project database and 2013 Korean Community Health Survey (CHS). Cases were defined as EMS-treated adult (18 year old and older) OHCA patients with presumed cardiac etiology collected at 27 emergency departments from January to December 2014. OHCA patients whose arrest occurred at nursing homes or clinics and cases with unknown information on DM were excluded. Four controls were matched to one case with strata including age, gender, and county from the Korean CHS database. Multivariable conditional logistic regression analysis was conducted to estimate the risk of DM and treatment modality on incidence of OHCA.

**Results:**

Total 1,386 OHCA patients and 5,544 community-based controls were analyzed. A total of 370 (26.7%) among cases and 860 (15.5%) among controls were diagnosed with DM. DM was associated with increasing risk of OHCA (AOR: 1.92 (1.65–2.24)). By DM treatment modality comparing with non-DM group, AOR (95% CI) was the highest in non-pharmacotherapy only group (4.65 (2.00–10.84)), followed by no treatment group (4.17 (2.91–5.96)), insulin group (2.69 (1.82–3.96)), and oral hypoglycemic agent group (1.55 (1.31–1.85)).

**Conclusion:**

DM increased the risk of OHCA, which was the highest in the non-pharmacotherapy group and decreased in magnitude with pharmacotherapy.

## Introduction

The overall public health burden of out-of-hospital cardiac arrest (OHCA) continues to increase due to simultaneous growth of cardiovascular comorbidities prevalence along with the aging population [1, 2]. Despite the development of intra-resuscitation and post-resuscitation care, the survival rate of OHCA remains low and neurological outcome is often impaired [3]. A better understanding of high risk groups of cardiac arrest especially in relation to cardiovascular comorbidities may provide the basis to develop and strategize prevention methods to decrease the burden of OHCA.

Diabetes mellitus (DM) is a well-established risk factor of cardiovascular comorbidities including coronary heart disease and ischemic stroke [4–6]. DM is also associated with increased risk for cardiac arrest by means of micro- and macro-vascular processes, elevating arrhythmogenic potential occurring as a result of diabetes-related autonomic neuropathy, increasing susceptibility to atherosclerosis and prevalence of atherogenic risk factors such as hypertension, dyslipidemia, obesity, and metabolic syndrome, hypercoagulable status secondary to DM, and diabetic cardiomyopathy [7–12].

The worldwide prevalence of DM has been dramatically increasing over time and is estimated to be about 8% among adults in 2014, where the heaviest burden lies in the population of more than 65 years of age [1, 13–15]. In addition, the prevalence of prediabetes with impaired glucose tolerance and/or impaired fasting glycaemia has also been on the rise, displaying a positive slope with increasing age. [1, 5]. Given the large number of diabetic patients worldwide, the absolute number of cardiac arrests attributable to DM is expected to be onerous and requires further investigation [1, 7].

Although a number of risk factors have been explicated in prior, whether and how the risk factors pose any different effect on OHCA incidence remains as a challenge to be understood. DM is a well-known independent risk factor for OHCA [9, 12, 16]; however, the variation of the risk of DM depending on the therapeutic methods on OHCA incidence is yet to be clarified. This study aimed to determine the risk of DM on OHCA incidence and to investigate whether the effects of DM on OHCA incidence were different across various treatment modality.

## Materials and Methods

### Study design and data source

This study was a case-control study using the Cardiac Arrest Pursuit Trial with Unique Registration and Epidemiologic Surveillance (CAPTURES) project database in Korea. The CAPTURES project was a prospective hospital-based patient cohort study, conducted from January to December 2014 at 27 emergency departments (EDs) (9 level 1 EDs and 18 level 2 EDs). This prospective multicenter project aimed to identify the risk factors of OHCA incidence and to evaluate determinants of the prognosis with long-term follow up. The CAPTURES project was subjected to patients with OHCA transported to the study EDs by emergency medical service (EMS) with resuscitation efforts (EMS-treated OHCA) and patients who had a presumed cardiac etiology as identified by emergency physicians in each ED. The project excluded the patients with terminal illness, under hospice care, with pregnancy, living alone or homeless without reliable information source, and a ‘Do Not Resuscitate’ card. OHCA patients of non-cardiac etiology, including trauma, drowning, poisoning, burn, asphyxia, or hanging, were also excluded.

The CAPTURES registry included patient’s socio-demographic information, health behaviors, past medical history, physical and emotional stress, EMS and ED information using Utstein template, laboratory test, cardiac examination, and short and long-term outcomes. Emergency physicians at each study ED collected the information using structured survey papers during a face-to-face interview with the patients’ family. Study coordinators at each study ED gathered laboratory test and cardiac examination results via medical record review and contacted the patients’ family members to conduct telephone interview to survey long-term outcomes after 6 and 12 months. Collected data were inputted and transferred to the central data server using EpiData version 3.1 (The EpiData Association, Denmark, Europe). All outliers or incorrect values were filtered by this data entry system.

The project quality management committee (QMC), which consisted of emergency physicians, epidemiologists, cardiologists, statistical experts, and medical record review experts, trained all study coordinators and investigators in each ED prior to joining the project. Additionally, the QMC gave feedback to study site coordinators on quality management of the CAPTURES data through monthly meetings. In order to obtain more comprehensive knowledge and maintain supervised information procurement, the study coordinators consulted the emergency physicians in the QMC for clarification when they were unable to objectively define a coding element.

Community-based controls were selected based on data from the 2013 Korean Community Health Survey (CHS) as conducted by the Korea CDC between September and November 2013. The Korean CHS is a nationwide, community-based, and cross-sectional household-level survey performed annually. The survey of 247 questionnaire items was conducted in 253 counties and aimed to gather reliable health-related information on acute and chronic diseases, health care utilization, health behaviors, quality of life, socio-environmental factors, and demographic information of responders.

A total of 220,258 participants completed the Korean CHS survey in 2013. The respondents were members of representatively selected households in 253 counties, which were sampled using probability proportional to size systematic sampling method and square root proportional allocation. All adult members who were 19 years old or older of the selected households were eligible and were recruited to complete the survey by each county health authority. An average of 920 adults in each county participated in the survey. All surveys were conducted by trained interviewers, who administered a face-to-face interview using structured survey papers and followed a strong quality management program and survey protocol [17].

### Study setting

The Korea EMS, which is a single-tiered and government-based system, provides a basic-to-intermediate level of ambulance services in sixteen provincial headquarters of the national fire department and supports a population of approximately 50 million [10]. Ambulances are operated by emergency medical technicians (EMTs) who are directed not to stop CPR without on-line medical control or declare death in field as well as during transport. Therefore, all EMS-assessed patients are transported to the ED [18].

The Ministry of Health and Welfare designated three levels to EDs by the resources and functional requirements according to the EMS Act; level 1 (n = 20) and level 2 (n = 130) EDs have more resources and better facilities for emergency care and must be staffed by emergency physicians 24 hours a day and 365 days a year, whereas level 3 EDs (n = 310) can be staffed by general physicians. Detailed information about EMS characteristics, OHCA protocols, ED characteristics has been reported previously [10, 19].

### Study participants

Cases were EMS-treated OHCA patients with presumed cardiac etiology who were transported to study participating EDs from January to December 2014 and were older than 18 years of age. OHCA patients whose arrest occurred at nursing homes or clinics and cases with unknown information on DM were excluded.

Controls were enrolled from the Korean CHS database. Controls were randomly matched within strata including age (10-year interval), gender, and county. Matching controls from the same county can ensure that controls are representative sample from the same source population. The ratio of controls to cases was 4:1; the ratio was determined in order to increase the statistical power of the study given the infrequent nature of the event.

### Measurements

Main exposure was physician-diagnosed DM prior to study enrollment and treatment method (pharmacotherapy or non-pharmacotherapy) as measured via survey. The type of DM or prevalence duration were not considered. DM treatment modality was classified into four categories: insulin, oral hypoglycemic agent, non-pharmacotherapy only, and no-treatment groups. Patients receiving combined therapy of insulin and oral hypoglycemic agent were classified as the insulin group.

The CAPTURES project used the same questionnaire for past medical history and health behaviors with the Korean CHS to ensure more comparable accuracy of information collected between cases and controls. Each past medical history for both cases and controls was recorded as “positive” if the condition was clinically recognized through physician diagnosis. We collected information on date of cardiac arrest, age, gender, county, past medical history including diagnosis of hypertension, and health behaviors including smoking (current smoker, ex-smoker, never-smoker, and unknown), alcohol (frequent alcohol consumption (more than twice per week), occasional alcohol consumption, never alcohol consumption, and unknown), physical exercise (vigorous, moderate, no, and unknown), sleeping hour (0–6, 6–8, over 8 hours, and unknown), and body mass index (BMI) (10.5–18.4, 18.5–24.9, over 25.0, and unknown).

### Statistical analysis

Demographic findings of OHCA case group and community control group were described. Continuous variables were compared using Wilcoxon rank sum test, and categorical variables were compared using chi-square test. For the missing variables for health behaviors (smoking (n = 274), alcohol (n = 1343), physical activity (n = 376), sleeping (n = 454), and BMI (n = 1039)), multiple imputations with multivariable proportional logistic regression models were conducted.

For the matched case-control dataset, multivariable conditional logistic regression analysis was conducted to estimate the effect of diagnosis and DM treatment modality on risk of OHCA, and to calculate the adjusted odds ratios (AORs) and 95% confidence intervals (CIs) after controlling for potential confounders as identified in directed acyclic graph (DAG) models including past medical history and health behaviors (smoking, alcohol, physical activity, sleeping hour, and body mass index). The variables included in the final model were examined for multicollinearity among potential factors. No collinearity among variables was detected. All statistical analysis was performed using SAS software, version 9.4 (SAS Institute Inc., Cary, NC, USA). P values are based on a two-sided significance level of 0.05.

### Ethics statements

The study complies with the Declaration of Helsinki and study protocol was approved by all Institutional Review Boards of 27 participating study institutions with waiver of informed consent. This study was financially supported by the Korea Centers for Disease Control and Prevention (CDC) (2013–2014).

## Results

### Demographic findings

**Fig 1:**
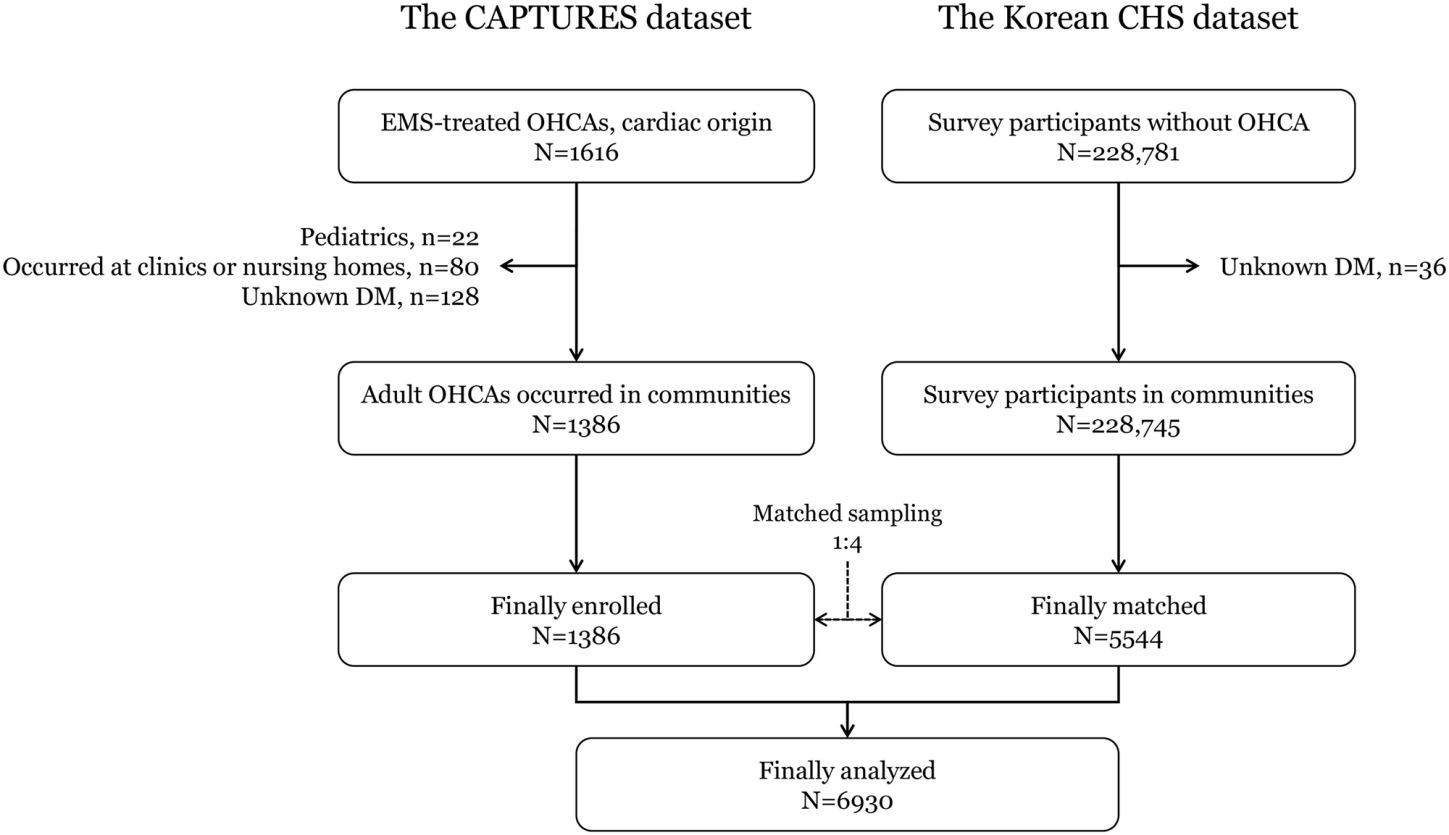
Study population flow. CAPTURES: Cardiac Arrest Pursuit Trial with Unique Registration and Epidemiologic Surveillance; CHS: Community Health Survey; EMS: emergency medical services; OHCA: out-of-hospital cardiac arrest; DM: diabetes mellitus.

Among 1,616 EMS-treated OHCAs during the study period, 1,386 patients were analyzed as case group excluding pediatrics (n = 22), patients whose arrest occurred at clinics or nursing homes (n = 80), and patients who had unknown information on DM (n = 128). For community control group, 5,544 subjects were selected within strata (case to control 1:4 matching ratio) from the 2013 Korean CHS database. A total of 1,616 matched case-control pairs were included in the study analysis (Fig 1).

The characteristics of the OHCA case and community control groups of the original and the imputed datasets are shown in S1 Table and Table 1. Of 1,386 OHCA patients with presumed cardiac origin, 370 (26.7%) were diagnosed with DM. Among them, 303 (81.9%) received treatment including insulin (n = 48, 13.0%), oral hypoglycemic agent (n = 244, 65.9%), and non-pharmacotherapy only (n = 11, 3.0%); whereas among 5,544 community controls, 860 (15.5%) were diagnosed with DM with 784 (91.2%) receiving treatment including insulin (n = 75, 8.7%), oral hypoglycemic agent (n = 696, 80.9%), and non-pharmacotherapy only (n = 13, 1.5%). We observed previous diagnosis of hypertension in 43.8% of OHCA patients and 39.2% of community controls (*p* <0.01).

**Table 1:** Demographics of out-of-hospital cardiac arrest cases and community controls.

	Total	OHCA cases	Community controls	
	N (%)	N (%)	N (%)	*p*-value
**Total**	6930	1386	5544	
**Gender**				1.00
**Female**	2195 (31.7)	439 (31.7)	1756 (31.7)	
**Male**	4735 (68.3)	947 (68.3)	3788 (68.3)	
**Age**				1.00
**19–29**	130 (1.9)	26 (1.9)	104 (1.9)	
**30–39**	320 (4.6)	64 (4.6)	256 (4.6)	
**40–49**	755 (10.9)	151 (10.9)	604 (10.9)	
**50–59**	1390 (20.1)	278 (20.1)	1112 (20.1)	
**60–69**	1140 (16.5)	228 (16.5)	912 (16.5)	
**70-**	3195 (46.1)	639 (46.1)	2556 (46.1)	
**Median (IQR)**	67 (53–74)	68 (53–78)	67 (53–74)	
**Past medical history**				
**Diabetes mellitus**				
**Diagnosis**	1230 (17.7)	370 (26.7)	860 (15.5)	<0.01
**Treatment**	1087 (15.7)	303 (21.9)	784 (14.1)	<0.01
**OHA**	940 (13.6)	244 (17.6)	696 (12.6)	<0.01
**Insulin**	123 (1.8)	48 (3.5)	75 (1.4)	<0.01
**Hypertension**				
**Diagnosis**	2781 (40.1)	607 (43.8)	2174 (39.2)	<0.01
**Treatment**	2543 (36.7)	522 (37.7)	2021 (36.5)	0.40
**Drug**	2532 (36.5)	518 (37.4)	2014 (36.3)	0.47
**Health behaviors**				
**Smoking**				<0.01
**Current**	1766 (25.5)	426 (30.7)	1340 (24.2)	
**Ex-smoker**	2069 (29.9)	283 (20.4)	1786 (32.2)	
**Never smoker**	3095 (44.7)	677 (48.8)	2418 (43.6)	
**Alcohol drink**				<0.01
**Frequent**	2294 (33.1)	387 (27.9)	1907 (34.4)	
**Occasional**	1516 (21.9)	304 (21.9)	1212 (21.9)	
**Never**	3120 (45.0)	695 (50.1)	2425 (43.7)	
**Physical activity**				<0.01
**Vigorous**	1364 (19.7)	142 (10.2)	1222 (22.0)	
**Moderate**	1142 (16.5)	241 (17.4)	901 (16.3)	
**No**	4424 (63.8)	1003 (72.4)	3421 (61.7)	
**Sleeping, hour**				<0.01
**0–6**	1371 (19.8)	205 (14.8)	1166 (21.0)	
**6–8**	3772 (54.4)	621 (44.8)	3151 (56.8)	
**8-**	1787 (25.8)	560 (40.4)	1227 (22.1)	
**Body mass index**				<0.01
**10.5–18.4**	408 (5.9)	114 (8.2)	294 (5.3)	
**18.5–24.9**	4748 (68.5)	897 (64.7)	3851 (69.5)	
**25.0-**	1774 (25.6)	375 (27.1)	1399 (25.2)	

OHCA: out-of-hospital cardiac arrest; IQR: interquartile range; OHA: oral hypoglycemic agent

The demographics according to prevalence of DM and treatment method are reported in Table 2. Diabetes patients treated with pharmacotherapy including oral hypoglycemic agent and insulin were more likely to be older, have hypertension, less physically active, and more obese, but less likely to be current smokers or frequent alcohol consumers than non-diabetes study subjects (all *p* <0.01). Among the diabetes patients, no treatment group was most likely to be physically active, current smoker, and frequent alcohol consumer than treatment groups.

**Table 2:** Demographics according to prevalence of diabetes mellitus and level of treatment.

	Total	DM (-)	DM and insulin	DM and OHA	DM and NPT	DM and no treatment	
	N (%)	N (%)	N (%)	N (%)	N (%)	N (%)	*p*-value
**Total**	6930	5700	123	940	24	143	
**Case-Control**							<0.01
**OHCA cases**	1386 (20.0)	1016 (17.8)	48 (39.0)	244 (26.0)	11 (45.8)	67 (46.9)	
**Community controls**	5544 (80.0)	4684 (82.2)	75 (61.0)	696 (74.0)	13 (54.2)	76 (53.1)	
**Gender**							0.45
**Female**	2195 (31.7)	1786 (31.3)	42 (34.1)	319 (33.9)	6 (25.0)	42 (29.4)	
**Male**	4735 (68.3)	3914 (68.7)	81 (65.9)	621 (66.1)	18 (75.0)	101 (70.6)	
**Age**							<0.01
**19–49**	1205 (17.4)	1153 (20.2)	3 (2.4)	36 (3.8)	0 (0.0)	13 (9.1)	
**50–59**	1390 (20.1)	1216 (21.3)	19 (15.4)	128 (13.6)	5 (20.8)	22 (15.4)	
**60–69**	1140 (16.5)	890 (15.6)	18 (14.6)	195 (20.7)	8 (33.3)	29 (20.3)	
**70-**	3195 (46.1)	2441 (42.8)	83 (67.5)	581 (61.8)	11 (45.8)	79 (55.2)	
**Median (IQR)**	67 (53–74)	65 (52–74)	72 (65–76)	71 (63–77)	69 (60–74)	70 (60–75)	
**Past medical history**							
**Hypertension**							
**Diagnosis**	2781 (40.1)	1974 (34.6)	74 (60.2)	631 (67.1)	21 (87.5)	81 (56.6)	<0.01
**Treatment**	2543 (36.7)	1804 (31.6)	69 (56.1)	611 (65.0)	21 (87.5)	38 (26.6)	<0.01
**Drug**	2532 (36.5)	1796 (31.5)	69 (56.1)	608 (64.7)	21 (87.5)	38 (26.6)	<0.01
**Health behaviors**							
**Smoking**							<0.01
**Current**	1766 (25.5)	1514 (26.6)	25 (20.3)	183 (19.5)	4 (16.7)	40 (28.0)	
**Ex-smoker**	2069 (29.9)	1657 (29.1)	34 (27.6)	332 (35.3)	10 (41.7)	36 (25.2)	
**Never smoker**	3095 (44.7)	2529 (44.4)	64 (52.0)	425 (45.2)	10 (41.7)	67 (46.9)	
**Alcohol drink**							<0.01
**Frequent**	2294 (33.1)	1990 (34.9)	26 (21.1)	224 (23.8)	7 (29.2)	47 (32.9)	
**Occasional**	1516 (21.9)	1268 (22.2)	22 (17.9)	197 (21.0)	6 (25.0)	23 (16.1)	
**Never**	3120 (45.0)	2442 (42.8)	75 (61.0)	519 (55.2)	11 (45.8)	73 (51.0)	
**Physical activity**							<0.01
**Vigorous**	1364 (19.7)	1191 (20.9)	10 (8.1)	127 (13.5)	4 (16.7)	32 (22.4)	
**Moderate**	1142 (16.5)	966 (16.9)	17 (13.8)	138 (14.7)	4 (16.7)	17 (11.9)	
**No**	4424 (63.8)	3543 (62.2)	96 (78.0)	675 (71.8)	16 (66.7)	94 (65.7)	
**Sleeping, hour**							<0.01
**0–6**	1371 (19.8)	1125 (19.7)	25 (20.3)	190 (20.2)	6 (25.0)	25 (17.5)	
**6–8**	3772 (54.4)	3171 (55.6)	62 (50.4)	454 (48.3)	14 (58.3)	71 (49.7)	
**8-**	1787 (25.8)	1404 (24.6)	36 (29.3)	296 (31.5)	4 (16.7)	47 (32.9)	
**Body mass index**							<0.01
**10.5–18.4**	408 (5.9)	338 (5.9)	7 (5.7)	48 (5.1)	0 (0.0)	15 (10.5)	
**18.5–24.9**	4748 (68.5)	3951 (69.3)	67 (54.5)	624 (66.4)	14 (58.3)	92 (64.3)	
**25.0-**	1774 (25.6)	1411 (24.8)	49 (39.8)	268 (28.5)	10 (41.7)	36 (25.2)	

DM: diabetes mellitus; NPT: non-pharmacotherapy only such as nutrition or exercise; OHA: oral hypoglycemic agent; OHCA: out-of-hospital cardiac arrest; IQR: interquartile range

*Non-pharmacotherapy included dietary intervention and physical activity prescription

### Main analysis

Results of multivariable conditional logistic regression models including AORs (95% CIs) for OHCA with diagnosis and treatment modality of DM are shown in Table 3. DM diagnosis was associated with increased risk for OHCA; the AOR (95% CI) for OHCA was 1.92 (1.65–2.24) after adjustment for other comorbidities and health behaviors. In terms of DM treatment, AOR (95% CI) was the highest in non-pharmacotherapy only group (4.65 (2.00–10.84)), followed by no treatment group (4.17 (2.91–5.96)), insulin group (2.69 (1.82–3.96)), and oral hypoglycemic agent group (1.55 (1.31–1.85)).

**Table 3:** Multivariable conditional logistic regression analysis of diagnosis and treatment of diabetes mellitus for out-of-hospital cardiac arrest incidence.

	OHCA cases/ Community controls	Unadjusted	Adjusted*
	*n/n*	OR (95% CI)	OR (95% CI)
**Model1: DM diagnosis**			
**No**	1016 / 4684	1.00	1.00
**Yes**	370 / 860	2.04 (1.77–2.36)	1.92 (1.65–2.24)
**Model2: DM treatment**			
**No DM**	1016 / 4684	1.00	1.00
**DM and no treatment**	67 / 76	4.17 (2.97–5.84)	4.17 (2.91–5.96)
**Non-pharmacotherapy only**	11 / 13	4.04 (1.80–9.05)	4.65 (2.00–10.84)
**Oral hypoglycemic agent**	244 / 696	1.66 (1.41–1.96)	1.55 (1.31–1.85)
**Insulin**	48 / 75	3.04 (2.10–4.40)	2.69 (1.82–3.96)

OHCA: out-of-hospital cardiac arrest; DM: diabetes mellitus; OR: odds ratio; CI: confidence interval

*Adjusted for hypertension, smoking, alcohol, physical activity, sleeping hour, and body mass index.

## Discussion

This case-control study, a pragmatic design for investigating relatively rare patient outcomes such as OHCA, found that DM increased the risk of OHCA and that the risk of DM on cardiac arrest exhibited various degrees of impact across different treatment methods. The magnitude of risk of DM on cardiac arrest was the highest for non-pharmacotherapy and lowest for oral hypoglycemic agent group after adjusting for past medical history and health behaviors. With the prevalence of DM steadily increasing temporally, intensive risk management and glycemic control should be highlighted in order to reduce the burden of DM as well as to lower adverse cardiovascular complications of DM.

Prevention of cardiovascular complication is crucial for diabetes patients. Intensive glycemic control and the HbA1c concentration management are strongly recommended to DM patients in order to reduce the incidence of adverse cardiovascular complications and mortality [4, 11, 12]. However, a previous study has found that glycemic control for diabetes patients significantly reduced the incidence of microvascular disease but had more limited effects on cardiovascular events including arrest [20]. In this study, DM increased the overall risk of cardiac arrest; however, the risk for OHCA in DM patients who were treated with oral hypoglycemic agent or insulin was observed to be smaller compared to no treatment or non-pharmacotherapy only groups. Our findings may be due to DM patients being treated with oral hypoglycemic agent or insulin complying with doctor recommendations and regimen adherence.

Lifestyle modification such as daily physical activity, weight management, blood pressure control, and lipid management is an additional treatment measure recommended for DM patients in order to prevent coronary and other macrovascular disease [21]. However, our study showed that diabetes patients were more likely to exhibit unhealthy behaviors (physical inactivity and obesity) and hypertension, although intensive risk management is currently recommended to decrease adverse complications of DM. Furthermore, non-pharmacotherapy only group was more likely to be obese than pharmacotherapy groups. Although a randomized controlled study has found that an intensive lifestyle intervention focusing on weight reduction did not reduce the rate of cardiovascular events among diabetes patients, the lifestyle prescriptions were associated with glycemic control and HbA1C reduction [22].

Along with the increasing temporal trend of DM prevalence, the population attributable risk of DM in cardiac arrest is also substantial [7]. In this study, DM was an independent risk factor for cardiac arrest, and diabetic patients with no treatment or non-pharmacotherapy only had a much higher risk compared to diabetic patients being treated with pharmacotherapy. Current recommendations to prevent cardiac arrest in relation to risk management including glucose and HbA1c management and intensive lifestyle prescriptions should be highlighted in order to decrease the burden of cardiac arrest among patients with DM.

### Limitations

This study has some limitations. First, the study design was a case-control study, not an intervention trial. There may be significant potential biases that were not controlled. We selected the control group from the same cohort of community residents who are theoretically the most preferable as controls (population at risk). However, there was possibility of misclassification for matching variables. Second, we measured DM by survey, and did not objectively confirm the diagnosis using blood tests. Survey questionnaire may be interpreted differently by survey responders, which can result in bias. Also, diagnosis of DM may have been under- or over-estimated, and it is possible that some patients had DM that was not diagnosed and therefore not recorded in the database. Third, this study classified DM as a single dichotomous variable, and types of DM, prevalence duration of DM, clinical manifestations, and presence of DM complications were not considered. We also did not survey why the patient selected certain treatment methods or obtain laboratory-confirmed values of glycemic control condition including hemoglobin A1c. The information was not available in our registry, and such factors may have yielded different effect sizes on the study outcome. Fourth, we didn’t gather information on past medical history of cardiovascular diseases including coronary artery disease and arrhythmia. A history of different cardiovascular diseases has been linked with diagnosis and therapeutic management of DM, as well as OHCA occurrence, which was not fully controlled for in this study; the lack of information may indicate residual confounding in the main analysis. Fifth, we made multiple imputations to process missing covariables. These factors might have not been adjusted fully, which may have induced measurement bias. Sixth, the timeframes for the case and control groups were slightly different; cases occurred during the calendar year of 2014 and controls were enrolled between September and November 2013. This difference is a potential, but likely minimal, biasing factor. Seventh, the investigators of the CAPTURES project were not blinded to the study hypothesis, which could have led to biased data collection from the data gatherers. Finally, the same questionnaire was used to obtain medical history and health behaviors from both cases and controls. This minimizes one potential bias but cannot eliminate the inherent source bias for the information on controls was presumably obtained from the controls themselves whereas the information about cases was mainly obtained from family members.

## Conclusions

Diabetes mellitus was an important risk factor for out-of-hospital cardiac arrest. The risk of DM on OHCA was the highest in the non-pharmacotherapy group, and the magnitude of risk was observed to be lower in the pharmacotherapy groups. Intensive risk management should be highlighted to reduce adverse cardiovascular complications of DM.

## Supporting Information

S1 Table Demographics of out-of-hospital cardiac arrest cases and community controls from the original dataset and health behaviors imputed dataset. (DOCX)
