# Supplementary material for: Risk of Diabetes Mellitus on Incidence of Out-of-Hospital Cardiac Arrests: A Case-Control Study
Source: PLoS One. 2016 Apr 22;11(4):e0154245. doi: 10.1371/journal.pone.0154245 (PMC4841534; doi:10.1371/journal.pone.0154245)
Supplement: S1 Table — (DOCX) [file pone.0154245.s001.docx]

S1 Table. Demographics of out-of-hospital cardiac arrest cases and community controls from the original dataset and health behaviors imputed dataset

|  | |  | Original dataset | | | | Imputed dataset* | | |
| --- | --- | --- | --- | --- | --- | --- | --- | --- | --- |
|  | |  | Total | OHCA cases | Community controls |  | Total | OHCA cases | Community controls |
|  | |  | N (%) | N (%) | N (%) | *p*-value | N (%) | N (%) | N (%) |
| Total |  | | 6930 | 1386 | 5544 |  | 6930 | 1386 | 5544 |
| Gender |  | |  |  |  | 1.00 |  |  |  |
|  | Female | | 2195 (31.7) | 439 (31.7) | 1756 (31.7) |  | 2195 (31.7) | 439 (31.7) | 1756 (31.7) |
|  | Male | | 4735 (68.3) | 947 (68.3) | 3788 (68.3) |  | 4735 (68.3) | 947 (68.3) | 3788 (68.3) |
| Age |  | |  |  |  | 1.00 |  |  |  |
|  | 19-29 | | 130 (1.9) | 26 (1.9) | 104 (1.9) |  | 130 (1.9) | 26 (1.9) | 104 (1.9) |
|  | 30-39 | | 320 (4.6) | 64 (4.6) | 256 (4.6) |  | 320 (4.6) | 64 (4.6) | 256 (4.6) |
|  | 40-49 | | 755 (10.9) | 151 (10.9) | 604 (10.9) |  | 755 (10.9) | 151 (10.9) | 604 (10.9) |
|  | 50-59 | | 1390 (20.1) | 278 (20.1) | 1112 (20.1) |  | 1390 (20.1) | 278 (20.1) | 1112 (20.1) |
|  | 60-69 | | 1140 (16.5) | 228 (16.5) | 912 (16.5) |  | 1140 (16.5) | 228 (16.5) | 912 (16.5) |
|  | 70- | | 3195 (46.1) | 639 (46.1) | 2556 (46.1) |  | 3195 (46.1) | 639 (46.1) | 2556 (46.1) |
|  | Median (IQR) | | 67 (53-74) | 68 (53-78) | 67 (53-74) |  | 67 (53-74) | 68 (53-78) | 67 (53-74) |
| Past medical history | | |  |  |  |  |  |  |  |
| Diabetes mellitus | | |  |  |  |  |  |  |  |
|  | Diagnosis | | 1230 (17.7) | 370 (26.7) | 860 (15.5) | <0.01 | 1230 (17.7) | 370 (26.7) | 860 (15.5) |
|  | Treatment | | 1087 (15.7) | 303 (21.9) | 784 (14.1) | <0.01 | 1087 (15.7) | 303 (21.9) | 784 (14.1) |
|  | OHA | | 940 (13.6) | 244 (17.6) | 696 (12.6) | <0.01 | 940 (13.6) | 244 (17.6) | 696 (12.6) |
|  | Insulin | | 123 (1.8) | 48 (3.5) | 75 (1.4) | <0.01 | 123 (1.8) | 48 (3.5) | 75 (1.4) |
| Hypertension | | |  |  |  |  |  |  |  |
|  | Diagnosis | | 2781 (40.1) | 607 (43.8) | 2174 (39.2) | <0.01 | 2781 (40.1) | 607 (43.8) | 2174 (39.2) |
|  | Treatment | | 2543 (36.7) | 522 (37.7) | 2021 (36.5) | 0.40 | 2543 (36.7) | 522 (37.7) | 2021 (36.5) |
|  | Drug | | 2532 (36.5) | 518 (37.4) | 2014 (36.3) | 0.47 | 2532 (36.5) | 518 (37.4) | 2014 (36.3) |
| Health behaviors | | |  |  |  |  |  |  |  |
| Smoking | | |  |  |  | <0.01 |  |  |  |
|  | Current | | 1687 (24.3) | 347 (25.0) | 1340 (24.2) |  | 1766 (25.5) | 426 (30.7) | 1340 (24.2) |
|  | Ex-smoker | | 1992 (28.7) | 206 (14.9) | 1786 (32.2) |  | 2069 (29.9) | 283 (20.4) | 1786 (32.2) |
|  | Never smoker | | 2977 (43.0) | 559 (40.3) | 2418 (43.6) |  | 3095 (44.7) | 677 (48.8) | 2418 (43.6) |
|  | Unknown | | 274 (4.0) | 274 (19.8) | 0 (0.0) |  | - |  |  |
| Alcohol drink | | |  |  |  | <0.01 |  |  |  |
|  | Frequent | | 1728 (24.9) | 237 (17.1) | 1491 (26.9) |  | 2294 (33.1) | 387 (27.9) | 1907 (34.4) |
|  | Occasional | | 1216 (17.5) | 209 (15.1) | 1007 (18.2) |  | 1516 (21.9) | 304 (21.9) | 1212 (21.9) |
|  | Never | | 2643 (38.1) | 556 (40.1) | 2087 (37.6) |  | 3120 (45.0) | 695 (50.1) | 2425 (43.7) |
|  | Unknown | | 1343 (19.4) | 384 (27.7) | 959 (17.3) |  | - |  |  |
| Physical activity | | |  |  |  | <0.01 |  |  |  |
|  | Vigorous | | 1264 (18.2) | 42 (3.0) | 1222 (22.0) |  | 1364 (19.7) | 142 (10.2) | 1222 (22.0) |
|  | Moderate | | 1074 (15.5) | 173 (12.5) | 901 (16.3) |  | 1142 (16.5) | 241 (17.4) | 901 (16.3) |
|  | No | | 4216 (60.8) | 795 (57.4) | 3421 (61.7) |  | 4424 (63.8) | 1003 (72.4) | 3421 (61.7) |
|  | Unknown | | 376 (5.4) | 376 (27.1) | 0 (0.0) |  | - |  |  |
| Sleeping, hour | | |  |  |  | <0.01 |  |  |  |
|  | 0-6 | | 1267 (18.3) | 101 (7.3) | 1166 (21.0) |  | 1371 (19.8) | 205 (14.8) | 1166 (21.0) |
|  | 6-8 | | 3526 (50.9) | 375 (27.1) | 3151 (56.8) |  | 3772 (54.4) | 621 (44.8) | 3151 (56.8) |
|  | 8- | | 1683 (24.3) | 458 (33.0) | 1225 (22.1) |  | 1787 (25.8) | 560 (40.4) | 1227 (22.1) |
|  | Unknown | | 454 (6.6) | 452 (32.6) | 2 (0.0) |  | - |  |  |
| Body mass index | | |  |  |  | <0.01 |  |  |  |
|  | 10.5-18.4 | | 326 (4.7) | 59 (4.3) | 267 (4.8) |  | 408 (5.9) | 114 (8.2) | 294 (5.3) |
|  | 18.5-24.9 | | 4055 (58.5) | 373 (26.9) | 3682 (66.4) |  | 4748 (68.5) | 897 (64.7) | 3851 (69.5) |
|  | 25.0- | | 1510 (21.8) | 168 (12.1) | 1342 (24.2) |  | 1774 (25.6) | 375 (27.1) | 1399 (25.2) |
|  | Unknown | | 1039 (15.0) | 786 (56.7) | 253 (4.6) |  | - |  |  |

OHCA: out-of-hospital cardiac arrest; IQR: interquartile range; OHA: oral hypoglycemic agent

*Imputed for missing variables of health behaviors with proportional logistic regression models
